# *Porphyromonas gingivalis* Differentially Modulates Cell Death Profile in Ox-LDL and TNF-α Pre-Treated Endothelial Cells

**DOI:** 10.1371/journal.pone.0154590

**Published:** 2016-04-28

**Authors:** Isaac Maximiliano Bugueno, Yacine Khelif, Narendra Seelam, David-Nicolas Morand, Henri Tenenbaum, Jean-Luc Davideau, Olivier Huck

**Affiliations:** 1 INSERM 1109 « Osteoarticular & Dental Regenerative Nanomedicine », Fédération de Médecine Translationnelle de Strasbourg (FMTS), Strasbourg, France; 2 Université de Strasbourg, Faculté de Chirurgie-dentaire, Department of Periodontology, Strasbourg, France; Federico II University, Naples, ITALY

## Abstract

**Objective:**

Clinical studies demonstrated a potential link between atherosclerosis and periodontitis. *Porphyromonas gingivalis* (*Pg*), one of the main periodontal pathogen, has been associated to atheromatous plaque worsening. However, synergism between infection and other endothelial stressors such as oxidized-LDL or TNF-α especially on endothelial cell (EC) death has not been investigated. This study aims to assess the role of *Pg* on EC death in an inflammatory context and to determine potential molecular pathways involved.

**Methods:**

Human umbilical vein ECs (HUVECs) were infected with *Pg* (MOI 100) or stimulated by its lipopolysaccharide (*Pg*-LPS) (1μg/ml) for 24 to 48 hours. Cell viability was measured with AlamarBlue test, type of cell death induced was assessed using Annexin V/propidium iodide staining. mRNA expression regarding caspase-1, -3, -9, Bcl-2, Bax-1 and Apaf-1 has been evaluated with RT-qPCR. Caspases enzymatic activity and concentration of APAF-1 protein were evaluated to confirm mRNA results.

**Results:**

*Pg* infection and *Pg*-LPS stimulation induced EC death. A cumulative effect has been observed in Ox-LDL pre-treated ECs infected or stimulated. This effect was not observed in TNF-α pre-treated cells. *Pg* infection promotes EC necrosis, however, in infected Ox-LDL pre-treated ECs, apoptosis was promoted. This effect was not observed in TNF-α pre-treated cells highlighting specificity of molecular pathways activated. Regarding mRNA expression, *Pg* increased expression of pro-apoptotic genes including caspases-1,-3,-9, Bax-1 and decreased expression of anti-apoptotic Bcl-2. In Ox-LDL pre-treated ECs, *Pg* increased significantly the expression of Apaf-1. These results were confirmed at the protein level.

**Conclusion:**

This study contributes to demonstrate that *Pg* and its *Pg*-LPS could exacerbate Ox-LDL and TNF-α induced endothelial injury through increase of EC death. Interestingly, molecular pathways are differentially modulated by the infection in function of the pre-stimulation.

## Introduction

Periodontal diseases are chronic inflammatory diseases affecting the tooth-supporting tissues. Pathogenesis of periodontitis is associated with dysbiosis of the periodontal microbiota. This dysbiosis is characterized by a shift from a symbiotic microbial community to a pathogenic one composed mainly of anaerobic bacteria resulting in alteration of the host-microbe cross-talk [1,2]. Periodontitis has been linked to several systemic diseases, especially atherosclerosis [3,4] while infection has been described as a potential mechanism involved in atherosclerosis worsening [4,5]. Interestingly, potential synergism has already been proposed for some risk factors of atherosclerosis, for instance periodontitis and obesity [6].

The role of infection in atherosclerosis has been proposed and several infective agents have been identified such as *Chlamydia pneumoniae*, *Helicobacter pylori* and *Porphyromonas gingivalis* (*Pg*) [3,7]. However, many aspects of the effects associated to infection, especially in an inflammatory context, remain unclear.

*Pg* is a gram-negative asaccharolytic bacterium implicated in periodontitis [1,7]. *Pg* is also considered as a keystone pathogen while it modulates gene and protein expression compromising immune function at the periodontal level [1,4]. Periodontal pathogens, including *Pg*, spread from periodontal pockets to general circulation and have been associated to atherosclerosis [4,8]. It has been detected in clinical human atheromatous plaque samples [8,9] and is able to worsen atheosclerosis in murine models [9,10]. Viable *Pg* has been detected in aorta of mice infected orally with *Pg* where it modulates innate immune response [10,11].

Endothelial cells (ECs) are key cells in vascular homeostasis and their dysfunction is associated with atherosclerotic process [11,12]. Due to their specific localization at the interface between inner part of the vessel and blood stream, ECs are under influence of several stressors such as bacterial pathogens including *Pg*. This bacterium, through its virulence factors such as lipopolysaccharide (*Pg*-LPS) is able to modify several molecular pathways associated to Toll-Like Receptors and innate immune response [12,13], activation of enzymes such as cathepsin B [13,14] and secretion of pro-inflammatory cytokines [14,15]. Interestingly, effects induced by the *Pg* infection in ECs appear to be strain-dependent [15,16].

ECs apoptosis has been observed in atheromatous plaque and may be involved in early phase of atherogenesis [16,17]. It increases vascular permeability, coagulation and promotes proliferation of smooth muscle cells [17,18]. Furthermore, non-phagocytosed apoptotic cells may undergo secondary necrosis contributing to vascular inflammation [18,19]. Several pathways have been described that are activated in ECs death, especially apoptosis, including caspase related pathways[19,20]. Apoptosis is a highly regulated mechanism activated through death receptors or perturbation of the mitochondria releasing cytochrome c that will induce pro-apoptotic factors activation [20,21]. Caspases are first synthesized as inactive pro-caspases that consist of a prodomain, which once initiated, activate a downstream or “effector” caspase such as caspase -3. Interestingly, the activation of caspase-9 is under the influence of the apoptosome complex constituted by apoptotic protease-activating factor-1 (Apaf-1). Apoptosome complex regulates apoptosis related cell death. However, its activation is under the control of several physiological mechanisms. Recently, apoptosome has been recognized as a potential therapeutic target in several diseases including diabetes and obesity [21,22]. Its implication in atherosclerosis has been recently proposed [22,23].

Several pro-atherogenic factors such as oxidized low-density lipoproteins (Ox-LDL) and TNF-α influence death of ECs, smooth muscle cells and macrophages, promoting necrotic core development [23,24]. Ox-LDL is an essential atherosclerotic risk factor that induce the expression of adhesion molecules, morphological changes of ECs [24,25] and apoptosis [22,25,26]. TNF-α is an inflammatory cytokine that worsen atherosclerotic development. This cytokine affects several vascular cell types, including ECs, and induces inflammatory, proliferative, cytostatic and cytotoxic effects. It has also been described as an inductor of ECs apoptosis [22,26,27]. Interestingly, some pathogens such as *Chlamydiae pneumoniae (C*.*pneumoniae)* modulate ECs death by promoting necrosis and reducing apoptosis[27,28].

The aim of our study was to evaluate the effects induced by *Pg* and its LPS on Ox-LDL and TNF- α induced cell death to assess the potential co-influence of atherosclerosis risk factors.

## Materials and Methods

### Bacterial culture

The *Pg* strain (ATCC 33277) was purchased from the American Type Culture Collection (ATCC, Manassas, VA, USA). Bacterial culture was performed under strict anaerobic conditions at 37°C in Brain-Heart Infusion medium supplemented with hemin (5mg/ml) and menadione (1mg/ml) purchased from Sigma (St. Louis, MO, USA). The day of the infection, bacterial culture was centrifuged and bacteria were washed twice with Phosphate Buffer Saline (PBS) and counted as previously described [16,28]. Heat-killed *Pg* (H*Pg*) was heated for 10 min at 85°C before the experimentation.

Commercial ultrapure *Pg*-LPS and *Escherichia coli*-LPS (*E*.*Coli*-LPS) were purchased from InvivoGen (San Diego, CA, USA).

### Cell culture

Human umbilical vein ECs (HUVECs) (C-12200, PromoCell, Heidelberg, Germany) were cultured in EGM2 medium (Promocell, Heidelberg, Germany) supplemented with 10% Fetal Bovine Serum at 37°C in a humidified atmosphere with 5% CO_2_. To investigate the effect of infection on cytotoxicity mediated by Ox-LDL and TNF-α, HUVECS were pre-treated 24h before challenge with either bacteria or LPS. For this purpose, 50μg/ml of Ox-LDL (Tebu-Bio, Le Perray en Yvelines, France) [27] or 10ng/ml TNF-α [29] (Tebu-Bio, Le Perray en Yvelines, France) has been added to cell culture medium.

### Infection of ECs with *Pg* and stimulation by LPS

Twenty-four hours before the experiment, 2x10^5^ cells were plated in each well of a 24-well plate. At the day of the experiment, HUVECs were washed twice with PBS and infected for 24 to 48h with *Pg* at a multiplicity of infection (MOI) of 100 bacteria/cell and stimulated by *Pg*-LPS (1μg/ml) and *E*.*Coli*-LPS (1μg/ml) for 24 to 48h.

### Cell viability

Cell viability was determined using colorimetric AlamarBlue test (Life Technologies). After 24 and 48h, 300 μl of incubation media were transferred to 96-well plates and measured at 570 and 600 nm in order to determine the percentage of AlamarBlue reduction.

### Live/Dead staining

The viability of HUVECs in all conditions was assessed using a fluorescence-based LIVE/DEAD^®^ assay (LIVE/DEAD® Cell Imaging Kit, Molecular Probes™, Invitrogen) at 24h. Cells were washed twice with phosphate-buffered saline (PBS; Fisher Scientific, Fair Lawn, NJ, USA) before staining. The staining solution consisted of 0.5 μL/mL calcein AM reagent and 2 μL/mL EthD-1 reagent mixed in 2 mL of PBS. Samples were incubated for 10 min and imaged using a 10x and 20x objective lens of a fluorescence microscope (Olympus BX53F, Tokyo, Japan) and filters for fluorescein and Texas Red for calcein and EthD-1 stains, and a digital CCD color imaging system (Microscope Digital Camera DP72; CellSens Entry^®^, Olympus, Tokyo, Japan).

### Type of cell death assessment

Apoptosis/necrosis ratio was analyzed using Annexin-V-FLUOS Staining Kit according to the manufacturer’s instructions (Roche Diagnostics, Meylan, France) at 24h. Cells were washed twice with PBS before staining. Cells were incubated with 100 μl of buffer solution, 5 μl of annexin V-FITC and 5 μl of propidium iodide (PI) for 15 min in the dark at room temperature. 50 μl of a solution of DAPI 200nM (Sigma-Aldrich Co., St Louis, MO, USA) was added for nuclear staining. Samples were imaged using a 10x and 20x objective lens of a fluorescence microscope (Olympus BX53F, Tokyo, Japan) and filters for fluorescein and Texas Red for calcein and EthD-1 stains, and a digital CCD color imaging system (Microscope Digital Camera DP72; CellSens Entry^®^, Olympus, Tokyo, Japan).

### RNA Isolation and Reverse Transcription

After cell lysis, total RNA was extracted using the High Pure RNA isolation kit (Roche Applied Science, Meylan, France) according to the manufacturer’s instructions. The extracted total RNA concentration was quantified using NanoDrop 1000 (Fischer Scientific, Illkirch, France). Reverse transcription was performed with the iScript Reverse Transcription Supermix (Bio-Rad Laboratories, Hercules, CA, USA) according to the manufacturer’s instructions.

### Quantitative Real-Time PCR Analysis

To quantify RNA expression, qPCR was performed on the cDNA samples. PCR amplification and analysis were achieved using the CFX Connect™ Real-Time PCR Detection System (Bio-rad, Miltry-Mory, France). Amplification reactions have been performed using iTaq Universal SYBR Green Supermix (Bio-rad, Miltry-Mory, France). Beta-actin was used as endogenous RNA control (housekeeping gene) in the samples. Primers sequences related to Bcl-2, Bax-1, Caspase-1, Caspase-3, Caspase-9 were purchased from Qiagen (Les Ulis, France) and sequence for Apaf-1 (3’-GTCTGCTGATGGTGCAAGGA-5’; 5’-GATGGCCCGTGTGGATTTC-3’) was synthesized (ThermoFischer, Saint-Aubin, France). The specificity of the reaction was controlled using melting curves analysis. The expression level was calculated using the comparative Ct method (2^−ΔΔCt^) after normalization to the housekeeping gene (β-actin). All PCR assays were performed in triplicate and results were represented by the mean values.

### Caspase activity fluorogenic assays

To determine caspase-1, -3 and -9 activity, cells were sonicated and lysates were incubated with 200 μL of substrate solution (20 mM HEPES, pH 7.4, 2 mM EDTA, 0.1% CHAPS, 5 mM DTT and 0.75 μM of caspase substrate) for 1 h at 37°C as previously described [30] [31]. The activities of caspase-1, -3 and -9 were calculated from the cleavage of the respective specific fluorogenic substrate (Ac-YVAD-AMC for caspase-1, AC-DEVD-AMC for caspase-3 and AC-LEHD-AMC for caspase-9) (Bachem, Bobendorf, Switzerland). Substrate cleavage was measured with a fluorescence spectrophotometer with excitation wavelength of 360 nm and emission at 460 nm. The data were calculated as fluorescence units/mg of total protein.

### Western blotting

In order to detect the protein level of Apaf-1, Western blot was performed. SDS-PAGE followed by immunoblotting were performed in conditions previously described [28]. Briefly, ECs collected from infection with *Pg* and from stimulation by *Pg*-LPS were lysed for 5 min on ice in 200 μl of ice-cold RIPA buffer (65 mM Tris–HCl, pH 7.4, 150mM NaCl, and 0.5% sodium deoxycholate) supplemented with phosphatase inhibitor cocktails I and II and a protease inhibitor cocktail (Sigma, Darmstadt, Germany). Lysates were centrifuged at 10,000 g at 4°C for 10min, and supernatants were collected for quantification using the Bradford protein assay (Bio-Rad, Hercules, CA, USA). To perform SDS-PAGE and immunoblotting, 25μg of proteins was used for each condition. The antibody against Apaf-1 (Rabbit) was purchased from ThermoFischer (Illkirch, France) (REF: PA5-19894) and against β-actin (Mouse) from Santa Cruz Biotechnology (Heidelberg, Germany) (REF:SC-130301). Secondary antibodies alkaline phospahatase conjugated (anti-mouse REF: A120-101AP; anti-rabbit REF: A90-116-AP) were purchased from Bethyl Laboratories (Montgomery, Texas, USA). All antibodies were used at the dilutions recommended by the manufacturer.

### Statistical analysis

All experiments were repeated at least 3 times and statistical analysis was performed using pairwise Anova test. Tukey’s post-hoc test was used to perform multiple comparisons. Data were analysed using PRISM 6.0 (GraphPad, La Jolla, CA, USA). Statistical significance was considered for *p*<0.05.

## Results

### Effect of Pg and its *Pg*-LPS on cell viability in ECs

**Fig 1:**
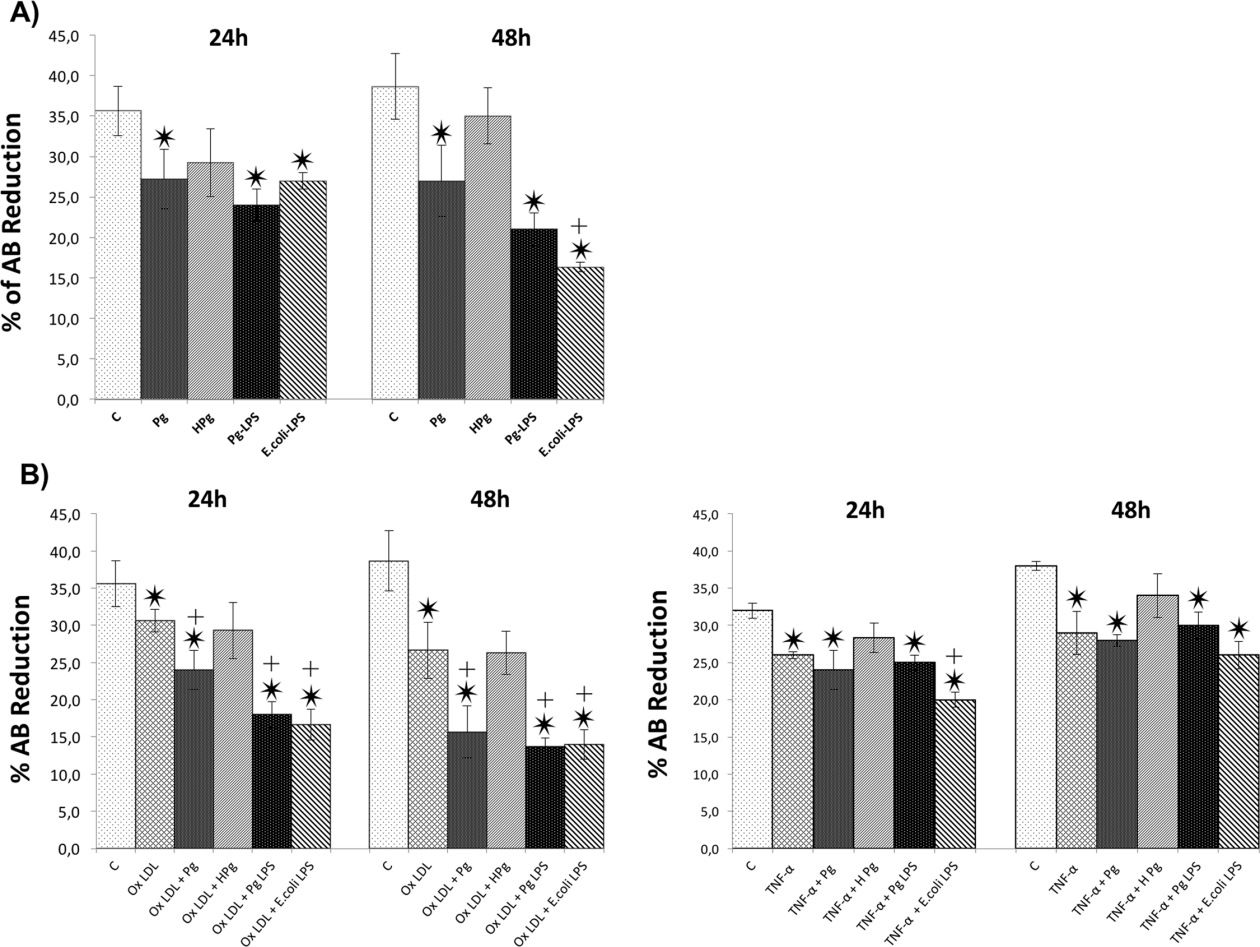
*Pg* ant its LPS increase ECs death. **(A)** Viability of HUVECs infected with *Pg* at a MOI of 100 or Heat-inactivated *Pg* (H*Pg*) and stimulated by *Pg*-LPS (1μg/ml) or *E*.*Coli*-LPS (1μg/ml) for 24h to 48h was measured using AlamarBlue test. **(B)** Viability of Ox-LDL (50μg/ml) and TNF- α (10ng/ml) pre-treated HUVECs with *Pg* at a MOI of 100 or Heat-inactivated *Pg* (H*Pg*) and stimulated by *Pg*-LPS (1μg/ml) or *E*.*Coli*-LPS (1μg/ml) for 24h to 48h. Data were expressed as mean ± SD. *: difference between non-stimulated/infected and stimulated/infected cells, *p* < 0.05, ┼: difference between non pre-treated/stimulated/infected and treated cells, *p* < 0.05.

**Fig 2:**
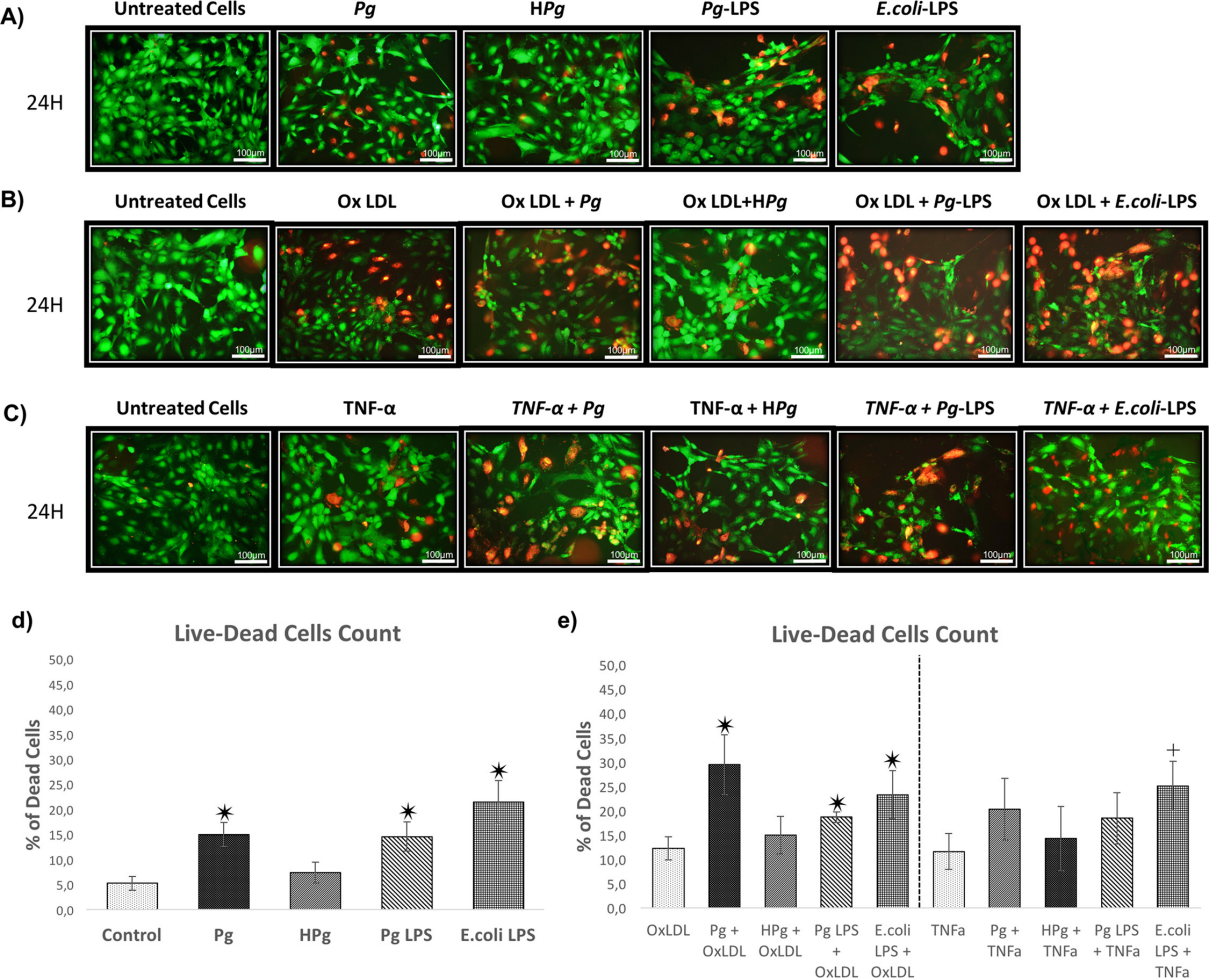
Qualitative evaluation of the EC death. **(A)** Viability of HUVECs infected with *Pg* at a MOI of 100 or Heat-inactivated *Pg* (H*Pg*) and stimulated by *Pg*-LPS (1μg/ml) or *E*.*Coli*-LPS (1μg/ml) at 24h. All different conditions have been evaluated quantitatively and qualitatively by Live-Dead staining assays. **(B)** Viability of Ox-LDL (50μg/ml) pre-treated HUVECs on cell cultures infected with *Pg* or H*Pg* at a MOI of 100 and stimulated by *Pg*-LPS (1μg/ml) or *E*.*Coli*-LPS (1μg/ml) at 24h. **(C)** Viability of TNF- α (10ng/ml) pre-treated HUVECs on cell cultures infected with *Pg* or H*Pg* at a MOI of 100 and stimulated by *Pg*-LPS (1μg/ml) or *E*.*Coli*-LPS (1μg/ml) at 24h. **(D)** Percentage of dead cells infected with *Pg* or H*Pg* and stimulated by *Pg*-LPS (1μg/ml) or *E*.*Coli*-LPS (1μg/ml) at 24h. **(E)** Percentage of dead cells in OxLDL qnd TNF- α pre-treated HUVECs. All images were acquired under fluorescence microscopy (in green: viable cells; in red: dead cells). All scale bars indicate 100 μm.

Cell viability was evaluated at 24 and 48h. At each time point, infection with *Pg* and stimulation by all tested LPS significantly decreased ECs metabolic activity (25% decrease for *Pg* infection versus control at 24h and 32% at 48h; 44% decrease for *Pg*-LPS stimulation versus control at 24h and 48h). No differences were observed between infection and *Pg*-LPS stimulation at 24 and 48h. Interestingly, ECs death was more important after *E*.*coli*-LPS stimulation at 48h. No effect on EC viability was observed with H*Pg* at 24 and 48h, highlighting a potential role of bacterial invasion in this process (Fig 1A). Regarding the impact of Ox-LDL and TNF-α, both stressors also significantly decreased cell viability at 24 and 48h (Fig 1B). For instance, Ox-LDL decreased EC viability up to 15% at 24h and 31% at 48h. This effect was amplified by *Pg* infection resulting in a decrease of EC viability up to 34% at 24h and 59% at 48h highlighting a cumulative effect between Ox-LDL and infection. This effect was not observed when TNF-α pre-treated cells were infected. A cumulative effect was only observed at 24h for *E*.*coli*-LPS in TNF-α pre-treated cells but not for *Pg*-LPS. These results were confirmed by Live/Dead staining (Fig 2).

### Modulation of type of cell death by *Pg*

**Fig 3:**
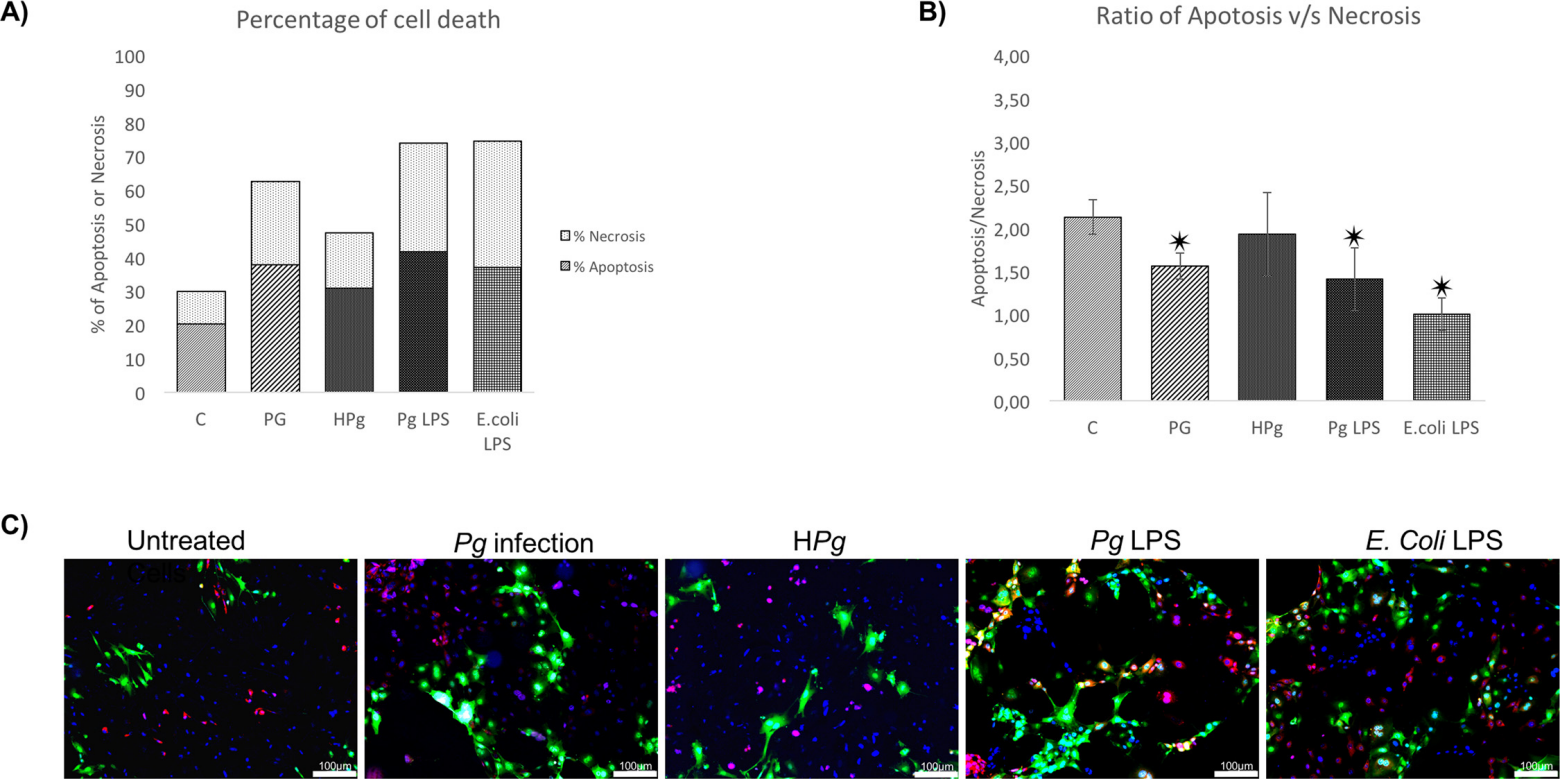
Infection of ECs leads to cell death mediated by apoptosis. **(A)** Percentage of cell death of ECs infected with *Pg* or Heat inactivated *Pg* (H*Pg*) at a MOI of 100 and stimulated by *Pg*-LPS (1μg/ml) or *E*.*Coli*-LPS (1μg/ml). Each percentage was calculated on account of total cells counted in triplicate for each experiment. **(B)** The apoptosis/necrosis ratio of ECs infected with *Pg* or Heat inactivated *Pg* (H*Pg*) at a MOI of 100 and stimulated by *Pg*-LPS (1μg/ml) or *E*.*Coli*-LPS (1μg/ml). Each value was calculated from the ratio between the total number of apoptotic cells and necrotic cells and for each count nine images were used of each experimentation. Data were expressed as mean ± SD. ✷: difference between non pre-treated/stimulated/infected and infected/stimulated cells, *p* < 0.05; **(C)** Infected and stimulated HUVECs cell death was evaluated for each condition qualitatively using Annexin V-IP staining at 24h (in green: Annexin V positive staining; in red: Iodure propidium positive staining; in blue: DAPI nuclear staining) Images were acquired under fluorescence microscopy (10x) after Annexin V-IP and DAPI staining for all previously described condition. All scale bars indicate 100 μm.

**Fig 4:**
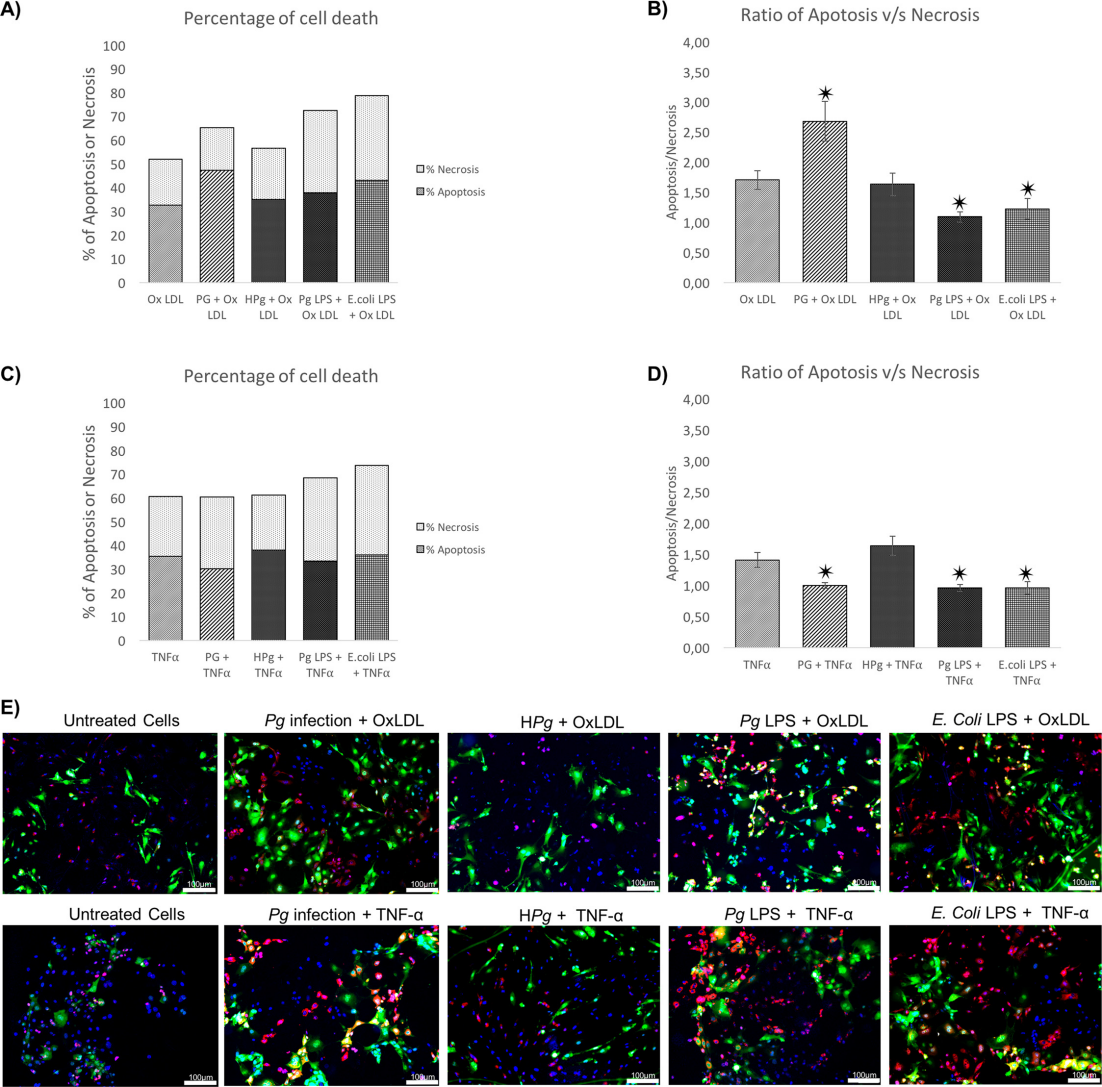
Pre-treatment of EC leads to different types of cell death induced by *Pg*. **(A)** Percentage of cell death of Ox-LDL (50μg/ml) pre-treated ECs infected with *Pg* at a MOI of 100 or Heat-inactivated *Pg* (H*Pg*) and stimulated by *Pg*-LPS (1μg/ml) or *E*.*Coli*-LPS (1μg/ml) for 24h. **(B)** The apoptosis/necrosis ratio of Ox-LDL (50μg/ml) pre-treated ECs with *Pg* at a MOI of 100 or Heat-inactivated *Pg* (H*Pg*) and stimulated by *Pg*-LPS (1μg/ml) or *E*.*Coli*-LPS (1μg/ml) for 24h. **(C)** Percentage of cell death of TNF-α (10ng/ml) pre-treated ECs infected with *Pg* at a MOI of 100 or Heat-inactivated *Pg* (H*Pg*) and stimulated by *Pg*-LPS (1μg/ml) or *E*.*Coli*-LPS (1μg/ml) for 24h. **(D)** The apoptosis/necrosis ratio of TNF- α (10ng/ml) pre-treated ECs infected with *Pg* at a MOI of 100 or Heat-inactivated *Pg* (H*Pg*) and stimulated by *Pg*-LPS (1μg/ml) or *E*.*Coli*-LPS (1μg/ml) for 24h. Data were expressed as mean ± SD. ✷: difference between non pre-treated/stimulated/infected and infected/stimulated cells, *p* < 0.05; **(E)** Infected and stimulated Ox-LDL (50μg/ml) and TNF- α (10ng/ml) pre-treated HUVECs cell death was evaluated for each condition qualitatively using Annexin V-IP staining at 24h and 48h (in green: Annexin V positive staining; in red: Iodure propidium positive staining; in blue: DAPI nuclear staining). Images were acquired under fluorescence microscopy (10x) after Annexin V-IP and DAPI staining for all previously described condition. All scale bars indicate 100 μm.

Type of cell death induced by each stressor was analyzed using Annexin V/ propidium iodide staining at 24h. Infection with *Pg* alone and stimulation by its LPS reduced apoptosis/necrosis ratio (Fig 3A and 3B). A specific effect was observed regarding the type of LPS used, *E*.*coli*-LPS seemed to induce more necrosis than *Pg*-LPS. In cells pre-treated by Ox-LDL and TNF-α, apoptosis was also the main type of cell death (Fig 4). Interestingly, an amplification of the apoptotic cell count was observed in Ox-LDL pre-treated cells infected with *Pg*. Such effect seemed to be independent of *Pg*-LPS, this virulence factor inducing mainly necrosis as it appeared with *E*.*coli*-LPS (Fig 4A and 4B). In TNF-α pre-treated cells, the same effect was not observed at 24h, where *Pg* infection induced an increase of necrosis in the same range as *Pg*-LPS and *E*.*coli*-LPS stimulation (Fig 4C and 4D).

### Modulation of cell death related mRNA expression

**Fig 5:**
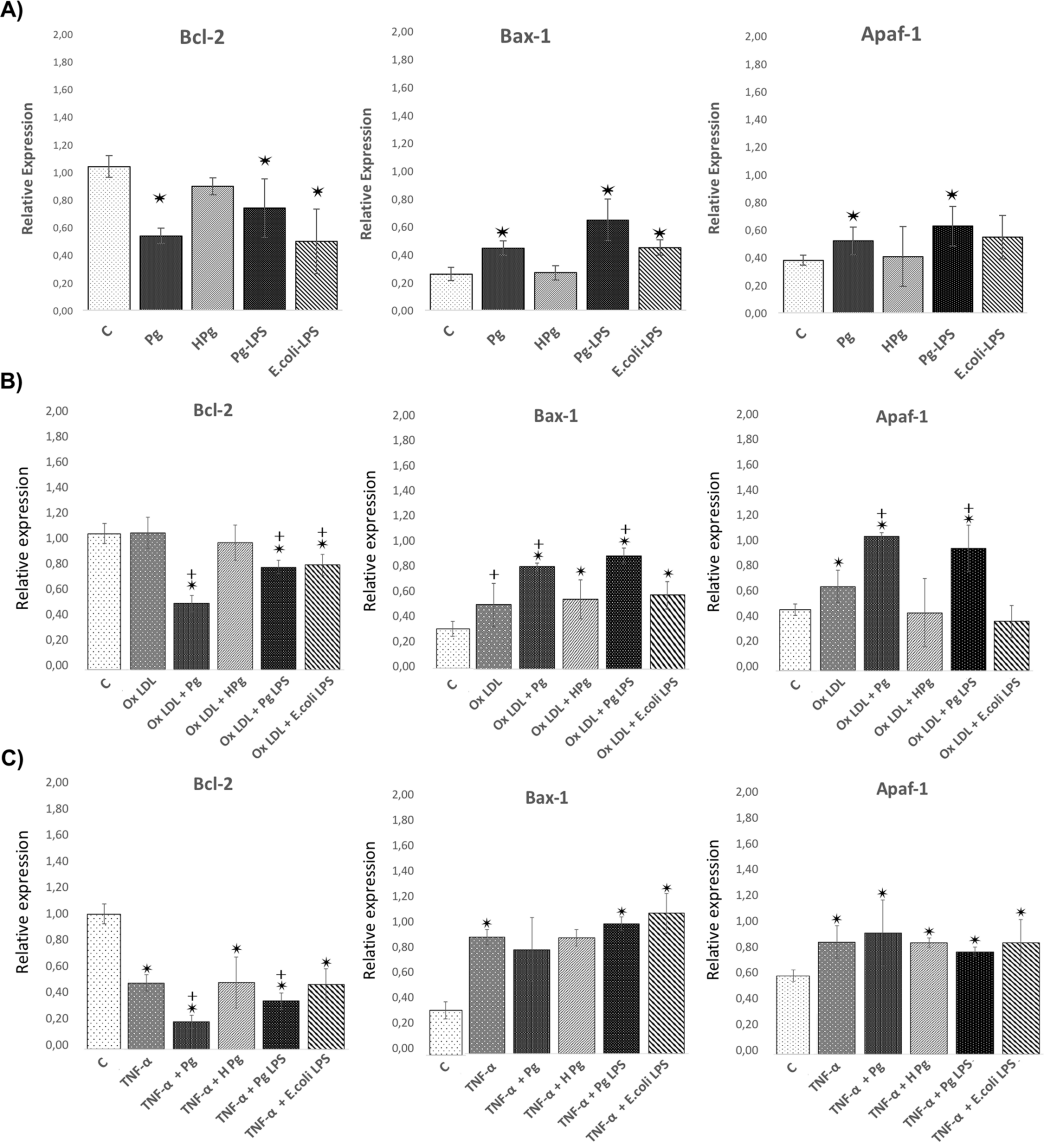
*Pg* and its LPS modulate the expression of EC death related gene expression. **(A)** Gene expression of Bcl-2, Bax-1, and Apaf-1 in HUVECs infected with *Pg* at a MOI of 100 or with heat inactivated *Pg* and stimulated by *Pg*-LPS (1μg/ml) or *E*.*Coli*-LPS (1μg/ml) at 24h. **(B)** Gene expression for the same described genes in Ox-LDL (50μg/ml) pre-treated HUVECs infected with *Pg* at a MOI of 100 or with heat inactivated *Pg* and stimulated by *Pg*-LPS (1μg/ml) or *E*.*Coli*-LPS (1μg/ml) at 24h. **(A)** Gene expression for the same described genes in TNF- α (10ng/ml) pre-treated HUVECs infected with *Pg* at a MOI of 100 or with heat inactivated *Pg* and stimulated by *Pg*-LPS (1μg/ml) or *E*.*Coli*-LPS (1μg/ml) at 24h. Data were expressed as mean ± SD. *: difference between non-stimulated/infected and stimulated/infected cells, *p* < 0.05, ┼: difference between non pre-treated/stimulated/infected and treated cells, *p* < 0.05.

**Fig 6:**
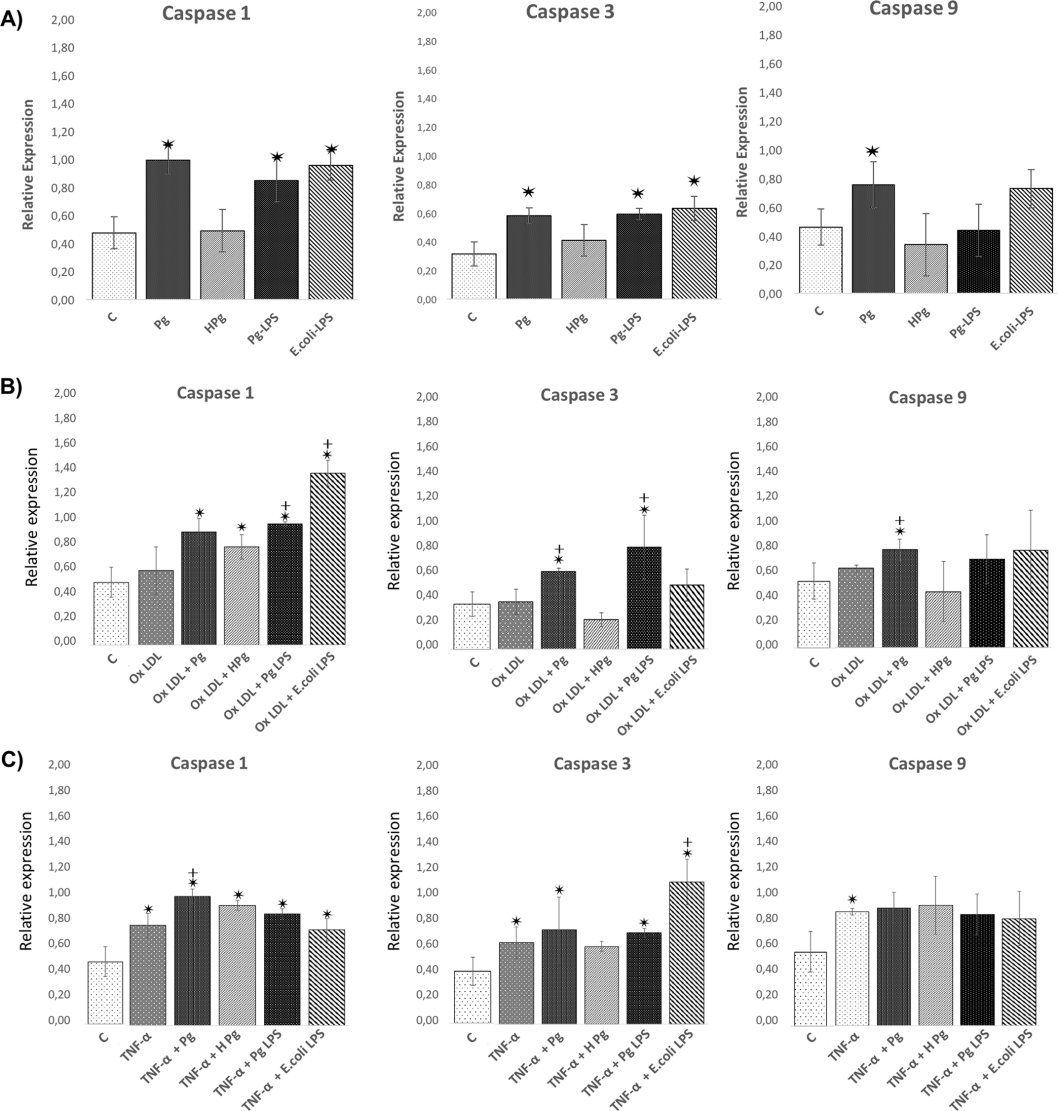
Differential modulation of the EC death related gene expression with *Pg* and its LPS in Ox-LDL and TNF-α pre-treated cells. **(A)** Gene expression of Caspase-1,-3 and -9 in HUVECs infected with *Pg* at a MOI of 100 or with heat inactivated *Pg* and stimulated by *Pg*-LPS (1μg/ml) or *E*.*Coli*-LPS (1μg/ml) at 24h. **(B)** Gene expression for the same described genes in Ox-LDL pre-treated HUVECs at 24h. **(C)** Gene Expression for the same described genes in TNF- α pre-treated HUVECs at 24h. Data were expressed as mean ± SD. *: difference between non-stimulated/infected and stimulated/infected cells, *p* < 0.05, ┼: difference between non pre-treated/stimulated/infected and treated cells, *p* < 0.05.

Cell death related gene expression was measured at 24h. Infection with *Pg* significantly increased expression of genes related to cell death including Bax-1, caspase-1, -3 and -9 and decreased expression of anti-apoptotic Bcl-2 (2-fold). An increase of Apaf-1 expression was already observed after *Pg* infection (1.45 fold) (Figs 5A and 6A). Interestingly, *Pg*-LPS induced similar modulation of gene expressions while *E*.*coli*-LPS did not modify significantly Apaf-1 related gene expression (Fig 5A). These results highlighted some cell death related pathways modulated during infection and may explain the decrease of cell viability induced by infection.

In Ox-LDL pre-treated cells, similar effects were observed regarding the expression of Bcl-2, Bax-1 and caspase-3. Infection with *Pg* increased significantly caspase-3, -9, Bax-1 and Apaf-1 expression and decreased Bcl-2 expression in comparison with Ox-LDL pre-treated cells (Figs 5B and 6B). This result corroborated the increase of apoptosis rate previously observed. Stimulation with *Pg*-LPS and *E*.*coli*-LPS decreased expression of Bcl-2 and increased expression of caspase-1. Only *Pg*-LPS increased expression of Bax-1 and caspase-3 (Figs 5B and 6B).

In TNF-α pre-treated cells, only Bcl-2 and caspase-1 were differentially expressed after *Pg* infection in comparison with TNF-α pre-treatment cells. Interestingly, *E*.*coli*-LPS significantly increased expression of caspase-3 (Figs 5C and 6C).

### Caspases activity

**Fig 7:**
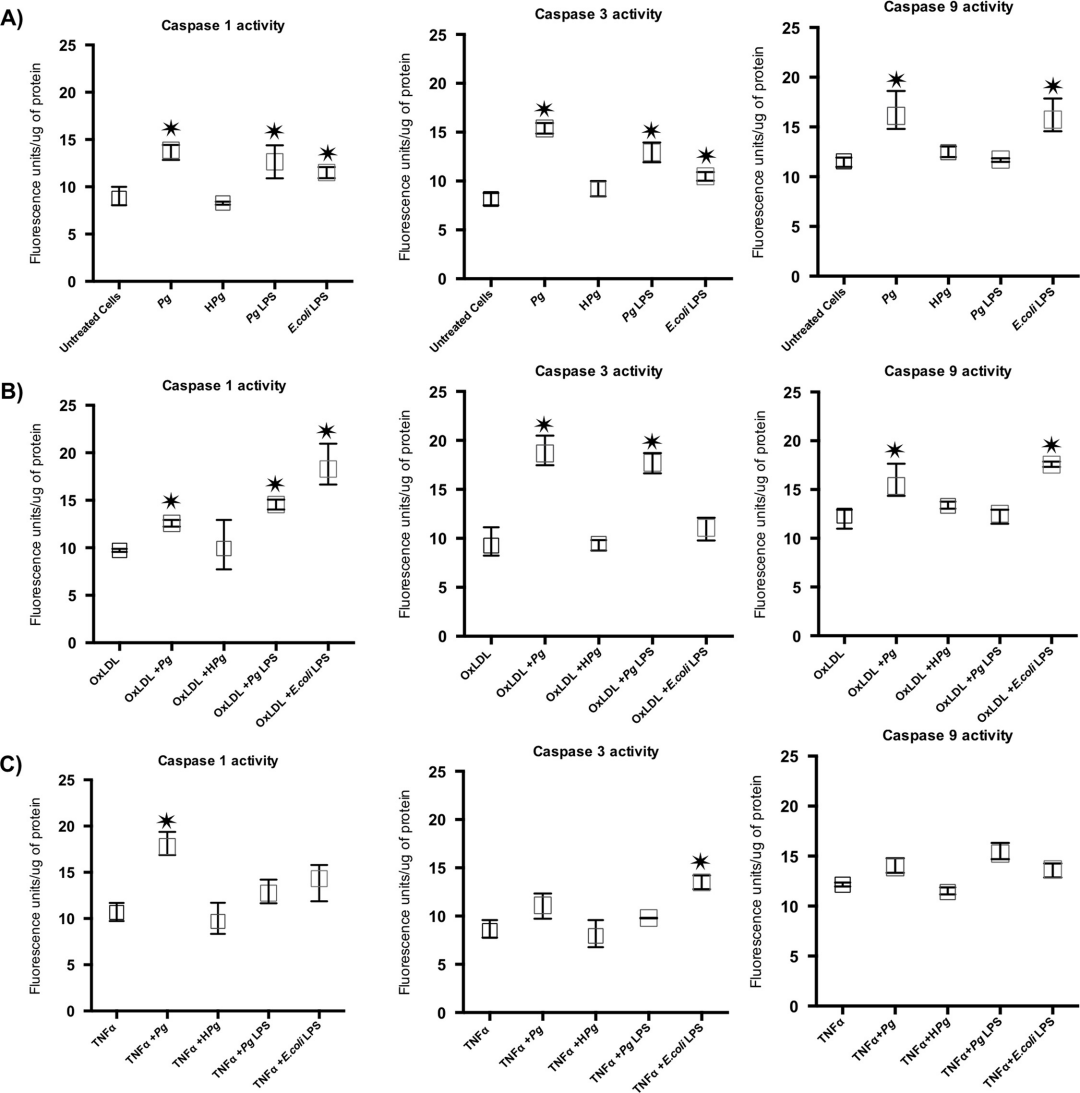
Differential modulation of the EC death related caspase activity after infection with *Pg* and its LPS in Ox-LDL and TNF-α pre-treated cells. **(A)** Enzymatic activity of Caspase-1, -3 and -9 in HUVECs infected with *Pg* at a MOI of 100 or with heat inactivated *Pg* and stimulated by *Pg*-LPS (1μg/ml) or *E*.*Coli*-LPS (1μg/ml) at 24h. **(B)** Enzymatic activity of Caspase-1, -3 and -9 in Ox-LDL pre-treated HUVECs at 24h. **(C)** Enzymatic activity of Caspase-1, -3 and -9 in in TNF- α pre-treated HUVECs at 24h. Data were expressed as mean ± SD. *: difference between non-stimulated/infected and stimulated/infected cells, *p* < 0.05.

To confirm the results observed at the mRNA level, enzymatic activities of the caspase-1, -3, and -9 were measured. Infection with *Pg* increased significantly the enzymatic activity of caspase-1, -3 and -9 (Fig 7A). Interestingly, Ox-LDL and TNF-α did not affect caspases activity in comparison with untreated cells (Fig 7). In Ox-LDL pre-treated ECs, a synergism between Ox-LDL and *Pg* infection has been highlighted regarding the activation of caspase-3 only (Fig 7B). Interestingly, in TNF-α pre-treated ECs, a synergy was observed regarding caspase-1 activity demonstrating the impact of one stressor on the potential molecular pathway activated by *Pg* (Fig 7C). A specific response was also observed, according to the pre-treatment with Ox-LDL or TNF-α, regarding the effects induced by *Pg*-LPS stimulation (Fig 7B and 7C).

### *Pg* and its LPS increase Apaf-1 protein expression

**Fig 8:**
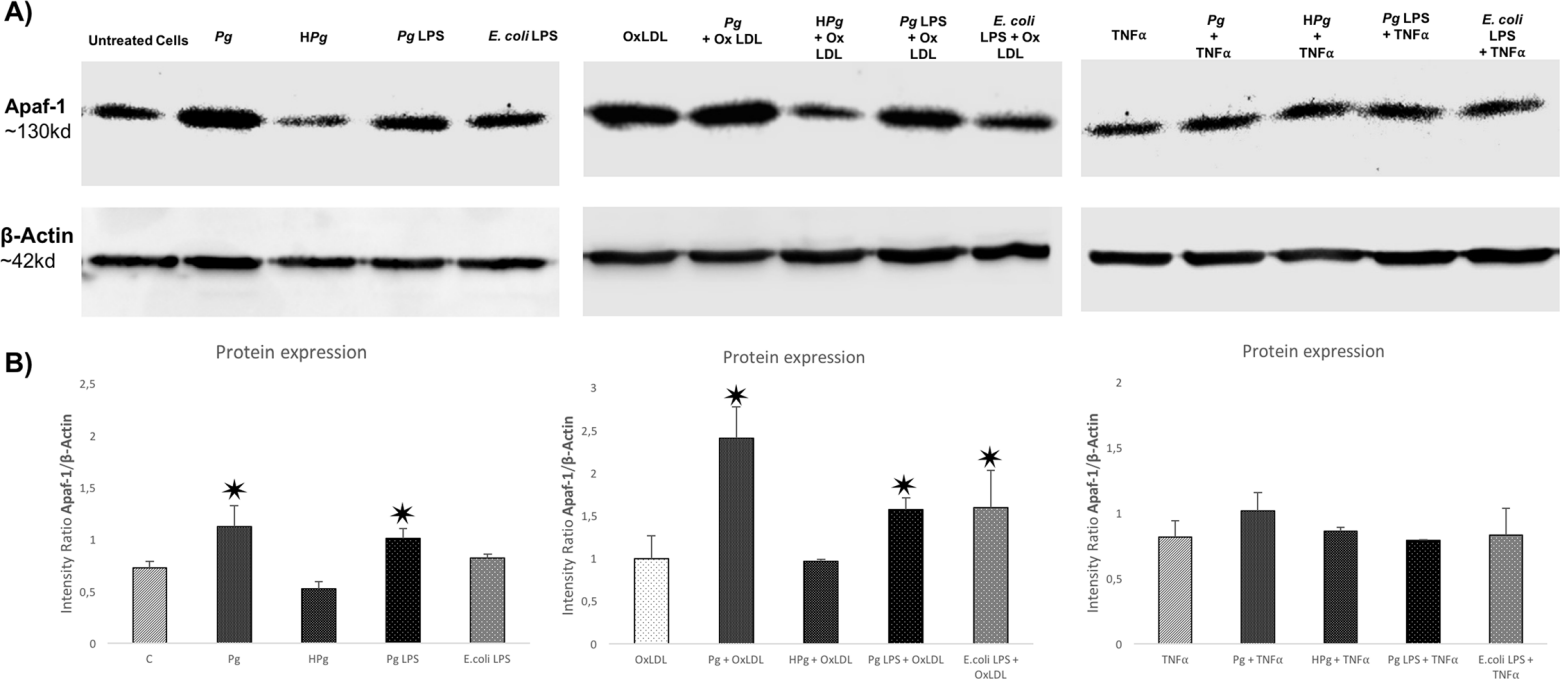
Differential modulation of Apaf-1 protein expression after infection with *Pg* and its LPS in Ox-LDL and TNF-α pre-treated cells. **(A)** Western blot analysis for Apaf-1 protein in HUVECs infected with *Pg* at a MOI of 100 or with H*Pg* and stimulated by *Pg*-LPS (1μg/ml) or *E*.*Coli*-LPS (1μg/ml) and pre-treated with Ox-LDL (50μg/ml) or TNF- α (10ng/ml) at 24h. **(B)** Density tracing was used to illustrate the quantitative differences in western blot analysis for Apaf-1 protein. Data were expressed as mean ± SD. *: difference between non-stimulated/infected and stimulated/infected cells, *p* < 0.05.

Regarding the effect on the Apaf-1 mRNA expression, an increase was observed with *Pg* infection especially in Ox-LDL pre-treated ECs (Fig 5B). This result was confirmed at the protein level (Fig 8). In Ox-LDL pre-treated ECs, *Pg* infection increased significantly the concentration of Apaf-1 (X2.5 vs Ox-LDL alone) (Fig 8B). Such effect was not observed in TNF-α pre-treated ECs as observed at the mRNA level (Fig 5C). These results were specific to live *Pg* as no modifications were observed when H*Pg* was used.

Regarding the *Pg*-LPS, a significant protein expression increase was observed following the stimulation in Ox-LDL pre-treated ECs (Fig 8).

## Discussion

The role of infection, especially with *Pg*, in atherosclerosis still needs to be clarified. Several risk factors have been identified for atherosclerosis including dyslipidemia [32] and systemic inflammation [33]. Some studies already demonstrated a synergism between risk factors as demonstrated for infection with *Pg* on endothelial injury in an obese mouse model [34]. However, mechanisms underlying this effect need to be determined.

In this study, we showed that infection with *Pg* increased EC death. As hypothesized, the pre-stimulation by another stressor, such as Ox-LDL amplified this cell mortality. However, no cumulative effect was observed after TNF-α pre-treatment. Interestingly, the type of cell death induced by *Pg* infection seemed to be under the influence of the inflammatory state of ECs, illustrating the cumulative influence of atherosclerotic risk factors on ECs survival.

ECs death was observed in early phases of atherogenesis [16,35] and several molecular risk factors could activate cell death related pathways. It has already been described that Ox-LDL, one of the most important atherosclerosis risk factors, was able to induce apoptosis in ECs in a concentration-dependent manner [27,35] and necrosis depending on Ox-LDL concentration and LDL oxidation level [5,27].

Infection with several periodontal pathogens, especially *Pg*, has been proposed as one of the potential mechanisms contributing to chronic inflammation of the atheromatous plaque [5,36] and also promoting lipid deposition within arterial wall [27,36]. Several bacteria have been described as being able to worsen atherosclerosis through induction of ECs death including *C*.*pneumoniae*, as demonstrated *in vitro* [27,37] and *in vivo* [37,38]. Regarding periodontal pathogens, especially *Pg*, it has been shown that this bacterium was able to mediate EC death in a dose-dependent manner [34,38] and to amplify endothelial injury through EC apoptosis induced by other molecules such as free fatty acids [27,34]. However, no data regarding the effects induced by *Pg* in the context of Ox-LDL or TNF-α pre-treated ECs were available. A cumulative effect between Ox-LDL and *Pg* infection on cell viability has been observed as demonstrated for *C*.*pneumoniae* [27,39]. *Pg*-LPS is one of the major virulence factors of *Pg* and endotoxemia has been considered to induce systemic inflammation contributing to atherosclerosis worsening [14,39]. In ECs, *Pg*-LPS has been described to induce secretion of several pro-inflammatory cytokines [13,14] and to activate different types of enzymes such as cathepsin B contributing to inflammation [13,40]. In our study, we showed that *Pg*-LPS decreased EC viability similar to *E*.*coli*-LPS. Interestingly, the impact on cell viability was higher in Ox-LDL and TNF-α pre-treated ECs. This synergism between *Pg*-LPS and Ox-LDL effect has already been observed in several cell types such as THP-1 [34,40], foam cells [41] and in hTERT-immortalized human umbilical vein endothelial cells (HuhT1) [34,42]. Nevertheless, its impact seemed to be cell-dependent as it was also showed that it inhibits apoptosis in epithelial cells [42,43] illustrating the complexity of cell responses to bacterial infection and endotoxemia.

In this study, we analysed the type of cell death induced by infection. Our results showed that cell death associated with *Pg* infection was both apoptosis and necrosis. Necrosis could be considered to be more pro-inflammatory than apoptosis. However, the significant increase of apoptosis was also detrimental to the organism [34,43]. Interestingly, the type of cell death induced was under the influence of EC pre-treatment. In Ox-LDL pre-treated ECs, *Pg* and its *Pg*-LPS significantly increased the apoptosis rate at 24h. This result was already observed in another EC model [34,44]. On the contrary, an increase of the necrosis rate was observed in TNF-α pre-treated ECs. This first observation illustrates the need of the use of complex cell models to mimic specifically the different processes involved in a vascular disease. In our study, type of cell death was determined by cells counting microscopically. A more precise evaluation could be achieve using flow-cytometry.

Apoptosis is considered as a programmed cell death. Recently, many other forms of programmed cell death have been described including pyroptosis. Pyroptosis is part of the host defense against infection and is dependent on the caspase-1 activation. This type of cell death is involved in several diseases characterized by inflammation [44,45]. *In vitro* evaluation of these processes seemed to be difficult and their roles have not been well defined *in vivo*. For instance, several features of pyroptosis seemed to overlap with apoptosis and pyroptosis and apoptosis shared a positive annexin V staining. Annexin V binds to phosphatidyl serine that is normally restricted to the inner leaflet of the cell membrane. During pyroptosis, pores are open in cell membrane allowing annexin V to enter the cell and stain [44,45]. In our study, *Pg* infection increased caspase-1 expression that could be considered as a potential trigger of pyroptotic cell death promoting inflammation [44,46].

Several pathways have been related to cell death, especially apoptosis. These pathways involved caspases that could be considered as the effectors and other complexes such as apoptosome that are activated after release of cytochrome c (cyt c) from the mitochondria. This process is under the influence of Bcl-2 and Bax [46]. In ECs, *Pg* increased cell death related gene expressions of Bax-1, caspase -1, -3, -9 and Apaf-1. To our knowledge, this is the first time that an influence of *Pg* on apoptosome related Apaf-1 gene expression was described. Furthermore, these modifications at the mRNA level were confirmed at the protein level. Caspase-1, -3 and -9 enzymatic activity was increased following the infection. Others studies showed influence of *Pg* on intrinsic apoptotic pathways, especially in epithelial cells [47,48].

Apaf-1 apoptosome is implicated in the intrinsic cell death pathway. Pro-apoptotic stimuli induce the release of cyt c that will act on this complex leading to activation of procaspase-9 and -3 and contributing to apoptosis [49]. Other bacteria have been described as being able to regulate apoptosis through Apaf-1/caspase-9 modulation [50]. Interestingly, in this study, Apaf-1 expression, at both mRNA and protein levels, was increased especially in Ox-LDL pre-treated ECs infected with *Pg* and also observed after stimulation by *Pg*-LPS highlighting a potential role for this virulence factor in this process and subsequently implication of TLR-4 related pathways [14].

In summary, this study contributes to demonstrate that *Pg* and its *Pg*-LPS could exacerbate Ox-LDL and TNF-α induced endothelial injury through increase of EC cell death. These results strengthen the hypothesis that periodontitis should be considered as a risk factor for atherosclerosis progression, especially in patients at-risk such as dyslipidemic and obese patients. This also highlights the need of strong collaboration between periodontists and cardiologists to diagnose and treat periodontal diseases to prevent atherosclerosis worsening.
